# Effect of partial substitution of fishmeal with insect meal (*Hermetia illucens*) on gut neuromuscular function in Gilthead sea bream (*Sparus aurata*)

**DOI:** 10.1038/s41598-021-01242-1

**Published:** 2021-11-08

**Authors:** Annalisa Bosi, Davide Banfi, Federico Moroni, Chiara Ceccotti, Maria Cecilia Giron, Micaela Antonini, Cristina Giaroni, Genciana Terova

**Affiliations:** 1 Department of Medicine and Surgery, University of Insubria, Via JH Dunant 5, Varese, Italy; 2 Department of Biotechnology and Life Sciences, University of Insubria, Via JH Dunant 3, Varese, Italy; 3 Department of Pharmaceutical and Pharmacological Sciences, University of Padova, Largo Meneghetti, Padova, Italy

**Keywords:** Neuroscience, Physiology, Zoology

## Abstract

Alternative nutrient sources to fishmeal for fish feed, such as insect meals, represent a promising sustainable supply. However, the consequences for fish digestive function have not been exhaustively investigated. In the present study we evaluated the effect of partial fishmeal substitution with 10% *Hermetia illucens* (Hi10) larvae meal on the neuromuscular function of proximal and distal intestine in gilthead sea bream. In animals fed with insect meal, weight and growth parameters were similar to controls fed with conventional fishmeal. In addition, no anomalies in intestinal gross morphology and no overt signs of inflammation were observed. The gastrointestinal transit was significantly reduced in Hi10 fed animals. In the proximal and distal intestine longitudinal muscle, Hi10 feeding downregulated the excitatory cholinergic and serotoninergic transmission. Sodium nitroprusside-induced inhibitory relaxations increased in the proximal intestine and decreased in the distal intestine after Hi10 meal. Changes in the excitatory and inhibitory components of peristalsis were associated with adaptive changes in the chemical coding of both proximal and distal intestine myenteric plexus. However, these neuromuscular function alterations were not associated with considerable variations in morphometric growth parameters, suggesting that 10% Hi meal may represent a tolerable alternative protein source for gilthead sea bream diets.

## Introduction

Aquaculture is continuously growing in the food chain industry and, as such, is facing serious pressure to provide sustainable outcomes^1^. Indeed, one of the main challenges of the aquaculture sector is to reach a more sustainable production chain. In the last few years, the use of fish oil and fish meal, as essential ingredients of common feeds containing highly nutritious and digestible proteins and lipids, has been gradually decreasing as a consequence of the high environmental impact and of the increased market price^2^. Among the emerging alternative nutrient sources for fish feed, insect meals represent a promising supply. *Hermetia illucens*, in particular, is considered a highly valuable feed ingredient as it can be easily cultured and reproduced in a controlled environment. In addition, its larval stage can grow on different organic substrates, thus revaluing waste materials, such as discarded vegetables^3^. Moreover, several reports have shown positive results for growth performance in cohorts of fish receiving partial replacement of fish meal with insect meals^4^.

However, only sparse data are available on the effects of insect meal diets on fish gut physiology. In fish, as in other organisms, the gastrointestinal tract is the principal organ system which has contact with the external environment and plays a fundamental role for body nutrition, growth, and survival. Indeed, in teleost fish, diet plays a fundamental role in the regulation of the digestive function owing to the different feeding habits, since they may be carnivorous, herbivorous or omnivorous, as well as to the anatomically different gut structures, such as the presence of pyloric caeca and, in some species, the absence of the stomach^5^. In fish, in analogy with other vertebrates, secretory, sensory and motor gut functions are controlled by intrinsic neuronal circuitries of the enteric nervous system (ENS). In mammals, the ENS is organized into ganglionated structures and interconnecting fibers forming two major plexuses, the submucosal plexus, which regulates intestinal secretion and absorption of nutrients, and the myenteric plexus, laying between the longitudinal and circular smooth muscle layers, which regulates motility^6,7^. At variance with the mammal ENS, the fish ENS does not display a well-organized structure of ganglia and interconnecting fibers, since neurons are distributed on the longitudinal muscle either as single cells or as aggregates of small groups of cells following nerve fiber bundles along the length of the gut^7,8^. Furthermore, in fish, the submucosal plexus is less well developed^7^. We have recently immunohistochemically characterized the major neurotransmitter pathways in the myenteric plexus of juvenile gilthead sea bream (*Sparus aurata*)^8^, which, along with the European sea bass (*Dicentrarchus labrax*), is a highly farmed fish species in Mediterranean aquaculture, with high economic impact^9^. In analogy to the ENS of other teleost fish^7^, in the juvenile gilthead sea bream proximal and distal intestine ENS we found neurons displaying morphological and chemical characteristics of the main neurotransmitter pathways involved in the regulation of peristalsis, although some differences were evidenced between the two regions, possibly due to the peculiar functions of each intestinal region^8^. In particular we could demonstrate the presence of the most important enteric neurotransmitters such as acetylcholine (ACh), substance P (SP), serotonin (5HT) and nitric oxide (NO) in different myenteric neuron populations, which showed the morphological characteristics of excitatory and inhibitory motor neurons, ascending and descending interneurons and primary sensory neurons involved in the peristaltic reflex^6,8^. Since changes in nutrient composition in the intestinal lumen may induce both short- and long-term changes in myenteric neurotransmitter expression, excitability, and viability, ultimately impacting gastrointestinal transit^10,11^, we aimed in this study to investigate possible adaptive changes in the neuromuscular component of the proximal and distal intestine of gilthead sea bream fed either with control fishmeal diet or with a diet containing 10% of *Hermetia illucens* larva meal. To this end, we evaluated the excitatory and inhibitory responses of the longitudinal muscle by means of in vitro organ bath assays. Furthermore, we evaluated whether substituting conventional normal fishmeal with insect meal could modify the distribution and abundance of ACh-, SP-, 5HT-, and NO-containing neurons in the myenteric plexus of the proximal and distal intestine of adult gilthead sea bream as well as the efficiency of the gastrointestinal transit in vivo.

## Results

### General macroscopic observations and histological assessment

At the end of the feeding trial gilthead sea bream receiving the Hi10 meal displayed a healthy phenotype similar to that of control (CTRL) animals with no statistically significant differences (*P* > 0.05) in growth performance between the groups (Final weight: CTRL tank1: 424.63 ± 16.86, CTRL tank2: 331.03 ± 21.40 CTRL tank3: 335.50 ± 16.21 and Hi10 tank1: 401.83 ± 25.85, Hi10 tank2: 369.63 ± 23.12 Hi10 tank3: 351.64 ± 22.69. Final length: CTRL tank1: 26.01 ± 0.41, CTRL tank 2 24.10 ± 0.31, CTRL tank 3: 24.48 ± 0.50 and Hi10 tank 1: 25.81 ± 0.60, Hi10 tank 2: 24.72 ± 0.50, Hi10 tank3: 24.26 ± 0.48. FCR (Feed Conversion Ratio) CTRL 1.67 ± 0.02 and Hi10 1.51 ± 0.18. SGR (Specific Growth Rate) CTRL: 0.42 ± 0.01 and Hi10: 0.43 ± 0.06 (data are expressed as mean ± SEM, n = 28 per tank). Upon gross visual inspection of gilthead sea bream, no major differences in the proximal and distal intestine obtained from control fishmeal-fed fish (CTRL) and fish fed with the *Hermetia illucens* 10% meal (Hi10) were observed between the two experimental groups.

**Figure 1:**
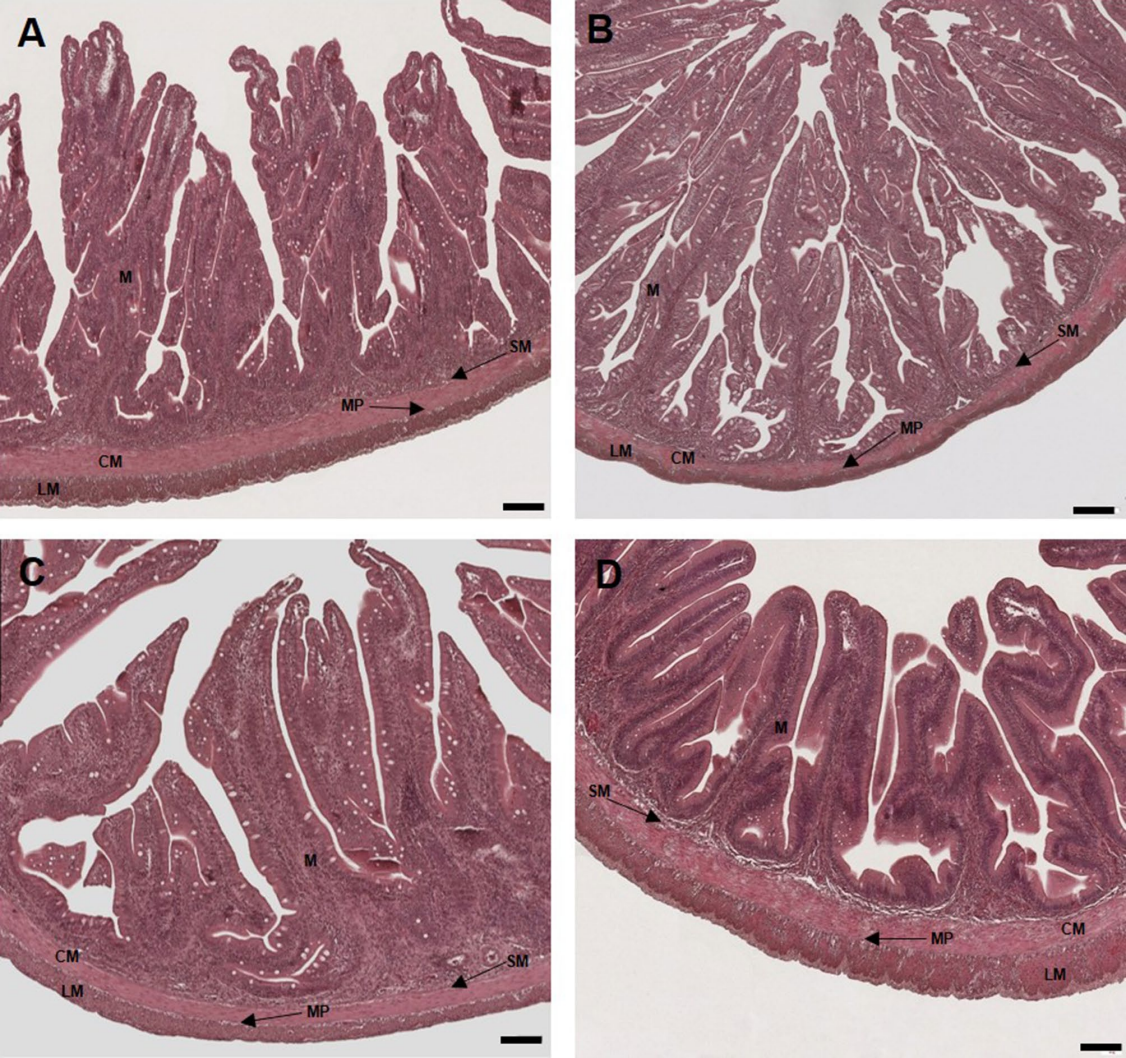
Standard hematoxylin–eosin (H&E) histochemical analysis of the proximal (Panels **A**, **B**) and distal (Panels **C**, **D**) gilthead sea bream intestine. 5 µm cross sections were obtained from proximal and distal paraffin-embedded intestine obtained from fishmeal fed fish (Panels **A** and **C**) and *Hermetia illucens* meal fed animals (panels **B** and **D**). M, mucosa; SM, submucosa; CM, circular muscle layer; LM, longitudinal muscle layer; MP, myenteric plexus. (Bar 100 µm).

Standard histological analysis of proximal and distal intestine cross sections obtained from Hi10 fish showed a well-organized and preserved tissue structure with no evident signs of damage as compared to preparations obtained from CTRL animals (Fig. 1, Panels A–D). At morphometric analysis of intestinal cross sections, however, some differences in preparations obtained from Hi10 animals were observed as compared to those obtained from CTRL animals. In the proximal intestine of Hi10 animals, villi density and width were significantly reduced as compared to values obtained in CTRL animals (*P* < 0.05 and *P* < 0.01, respectively) (Fig. 1 Panels A–B, and Supplementary Table S1). Both in the proximal and distal intestine, the architecture of the submucosal layer was not affected by *Hermetia illucens* meal (Fig. 1 Panels A–D, and Supplementary Table S1).

**Figure 2:**
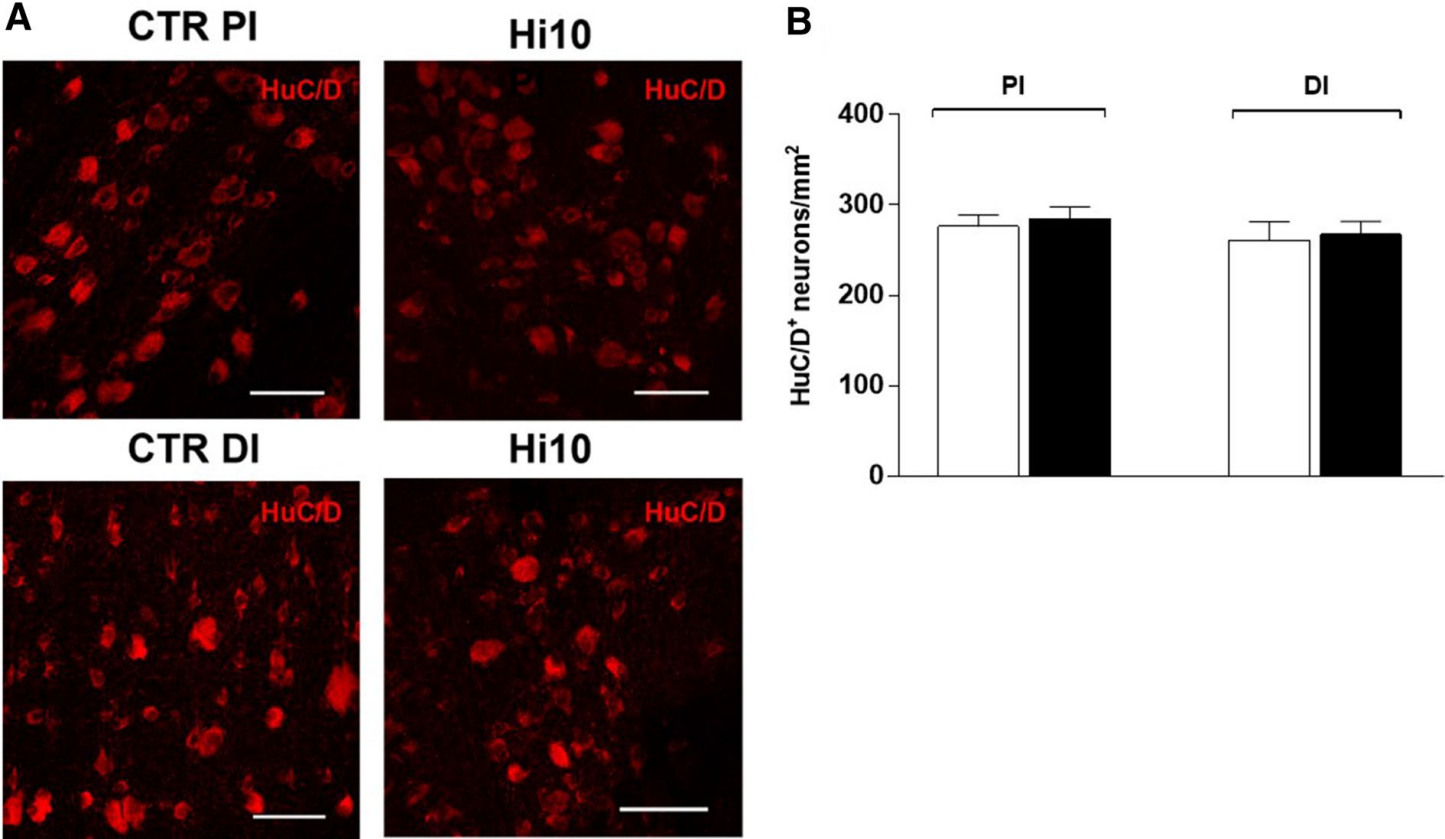
Density of myenteric neurons in longitudinal muscle myenteric plexus (LMMP) whole mounts of the proximal and distal gilthead sea bream. (**A**) HuC/D staining in LMMP preparations of the proximal and distal gilthead sea bream intestine obtained from fishmeal fed (CTRL) and *Hermetia illucens* 10% (Hi10) fed fish. Bars: 50 µm. (**B**) Number of myenteric neurons per mm^2^ staining for HuC/D in gilthead sea bream proximal (PI) and distal (DI) intestine obtained from CTRL (empty bars) and Hi10 (black bars) animals. Values are given as mean ± SEM (n = 5 fish).

In the distal intestine, the thickness of the smooth muscle layer was significantly greater than that in CTRL animals (*P* < 0.001). This enhancement was observed in both the longitudinal and circular layers and was particularly prominent in the circular layer (*P* < 0.05 and *P* < 0.001, respectively) (Fig. 1 Panels C–D, and Supplementary Table S1). In the proximal intestine, the *muscularis propria* also tended to be thicker after feeding with the Hi10 meal, however, this change did not reach statistical significance. Myenteric neurons were found between the circular and longitudinal muscle of the *muscularis propria* of both regions by immunofluorescence staining with the pan neuronal marker HuC/D (Fig. 2, panel A). Myenteric neurons appeared as either single neuronal cells or small aggregates, consisting of 3–8 cells distributed over the longitudinal layer (Fig. 2, panel A). HuC/D immunoreactivity distribution was cytosolic and did not display signs of neuronal injury, such as nuclear translocation. In the proximal and distal intestine of both CTRL and Hi10 fish, the number of myenteric neurons, normalized per area (mm^2^), was similar (proximal CTRL: 276.4 ± 12.87; proximal Hi10: 284.5 ± 13.6; distal CTRL: 261.1 ± 20.50; distal Hi10: 267.9 ± 14.17, N = 5; (data expressed as mean ± SEM) (Fig. 2 panel B).

### Gastrointestinal transit in CTRL and in *Hermetia illucens* fed gilthead sea bream

**Figure 3:**
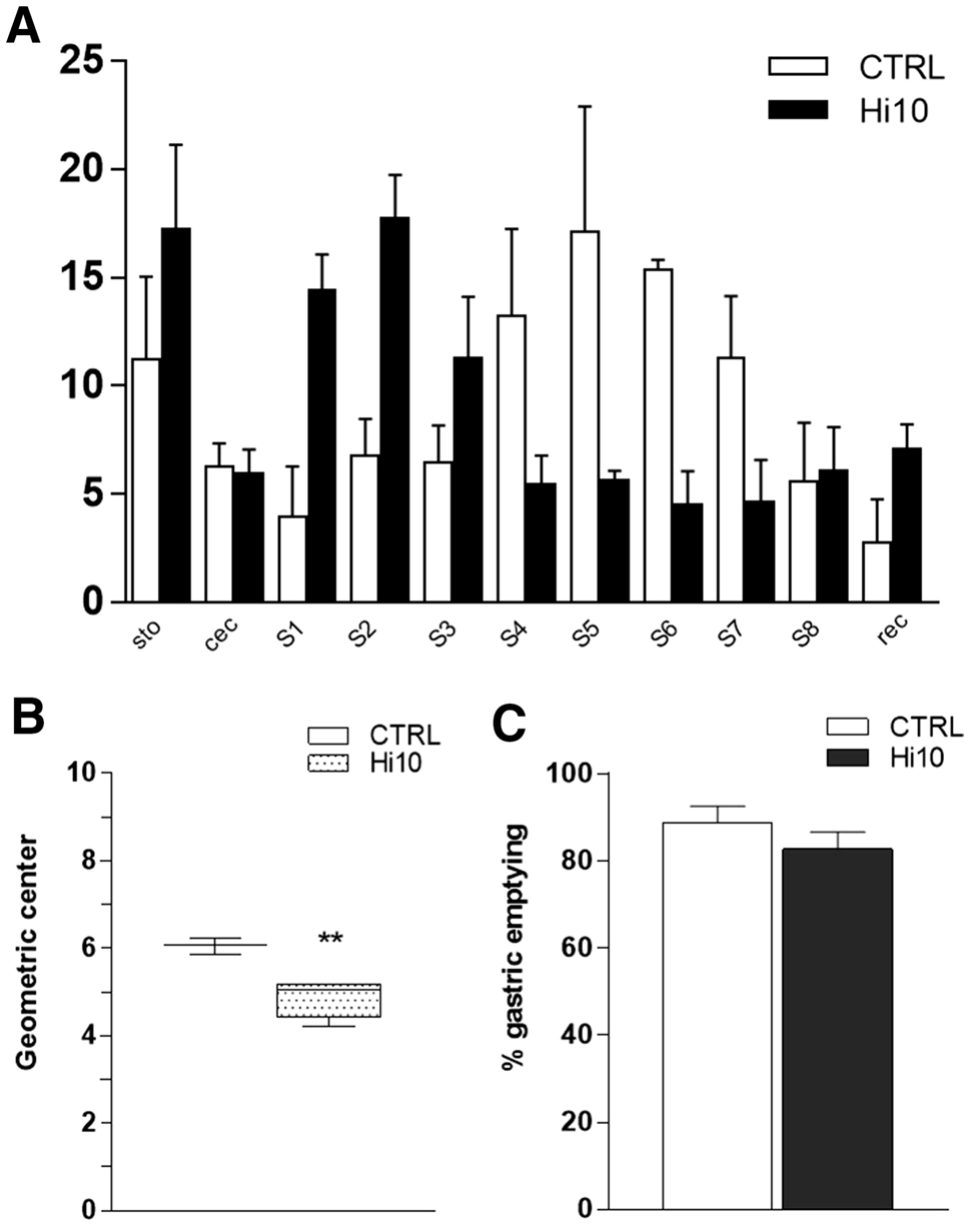
*Hermetia illucens* meal influences the efficiency of the gastrointestinal transit in gilthead sea bream. (**A**) Percentage of non-absorbable FITC-dextran 250 kDa distribution along the gastrointestinal tract consisting of stomach (sto), pyloric caeca (cec), proximal intestine (S1–S3), median intestine (S4-S6), distal intestine (S7-S8) and rectum (rec). (**B**) Geometric center of non-absorbable FITC-dextran distribution. (**C**) Percentage of gastric emptying in the different experimental groups. Data are reported as mean ± SEM for panels (**A**) and (**C**) and as median, minimum, maximum, upper and lower quartiles for panel (**B**). **P < 0.01 versus CTRL by unpaired Student’s t test. n = 5 fish/experimental group.

Partial substitution of fishmeal with *Hermetia illucens* meal affected gilthead sea bream gastrointestinal motor function, as suggested by the slower gastrointestinal transit as compared to CTRL animals (Fig. 3, panel A). This is reflected by the significant (*P* < 0.01) reduction in the geometric center (GC) of fluorescence distribution in Hi10 animals with respect to the value obtained in CTRL animals (Fig. 3, panel B). Gastric emptying was not significantly modified by *Hermetia illucens* meal as compared to CTRL diet (Fig. 3, panel C).

### *Hermetia illucens* meal influences the excitatory neuromuscular contractility of the proximal and distal intestine in gilthead sea bream

The longitudinal muscle of the proximal (PI) and distal (DI) intestine of CTRL animals showed spontaneous movements consisting of regular pendular contractions, which were significantly higher (*P* < 0.01) in the distal than in the proximal intestine (Supplementary Table S2). In both intestinal regions, the amplitude of spontaneous contraction was significantly reduced after feeding with *Hermetia illucens* meal as compared to the relevant CTRL value (PI: *P* < 0.001; DI: *P* < 0.05). The frequency of spontaneous basal contractions was significantly higher in the proximal intestine than values obtained in the distal intestine (*P* < 0.01) (Supplementary Table S2). In both intestinal regions, *Hermetia illucens* meal significantly reduced the frequency of spontaneous contractions with respect to the relevant CTRL value (*P* < 0.05 for both PI and DI; Supplementary Table S2).

**Figure 4:**
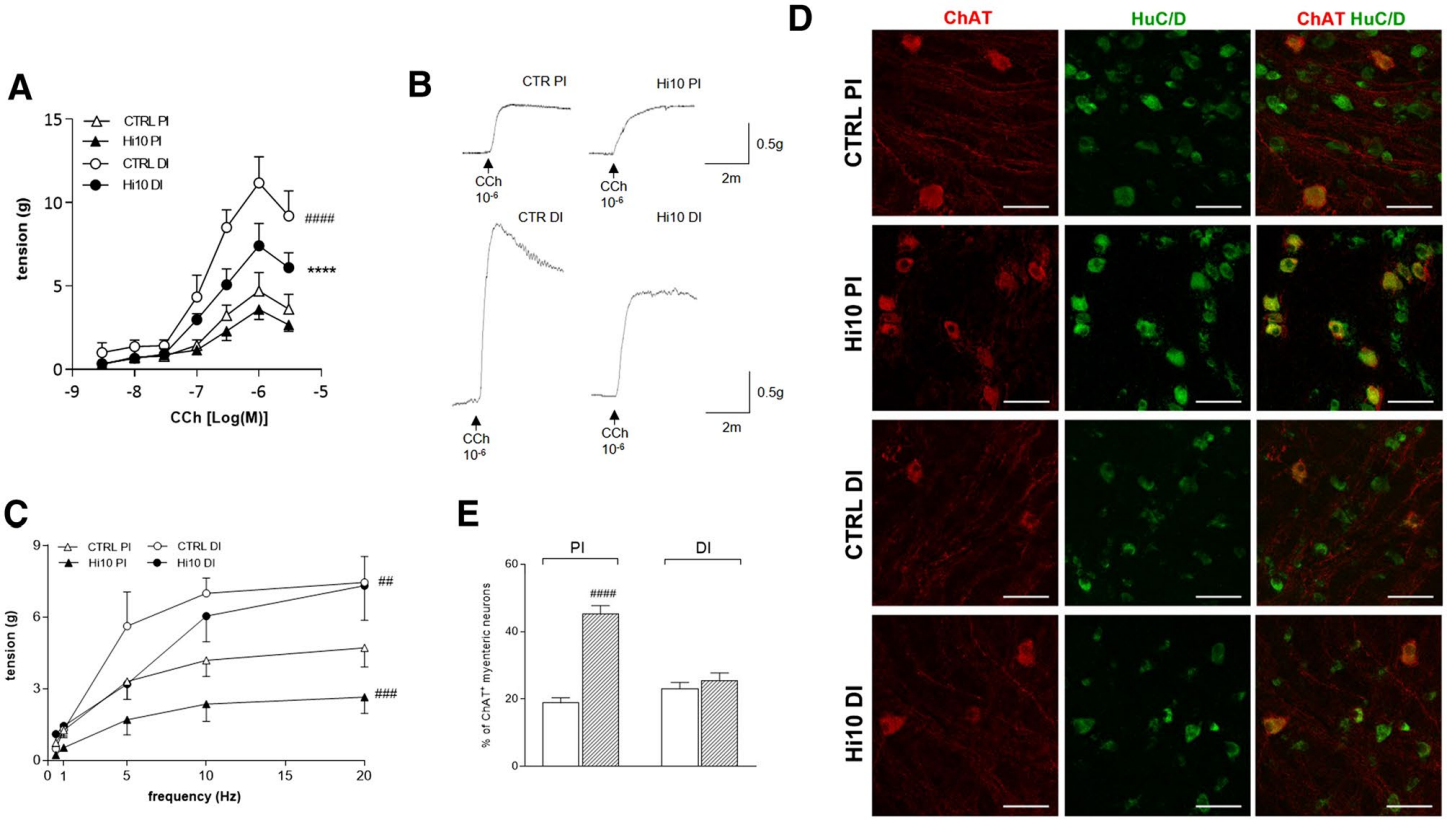
*Hermetia illucens* meal influences excitatory contractility in the proximal and distal intestine of the gilthead sea bream. (**A**) Concentration–response curves to carbachol (CCh) of isolated proximal (PI) and distal (DI) intestinal segments obtained from animals fed with a fishmeal standard diet (CTRL) and with a diet containing 10% *Hermetia illucens* (Hi10) meal. (**B**) Tracing displaying the excitatory effect of CCh 1 µM on basal tone of the proximal and distal gilthead sea bream obtained from CTRL and Hi10 animals. (**C**) Neuromuscular excitatory responses induced by EFS (0.5–20 Hz) in isolated proximal and distal intestine preparations obtained from the different experimental groups. N = 5 fish/group. (**D**) Representative confocal photomicrographs showing the distribution of ChAT (red, marker for cholinergic neurons) and HuC/D (green, pan-neuronal marker) in the different experimental groups. Bars: 50 μm (**E**) percentage of ChAT^+^ myenteric neurons in LMMP preparations of the proximal (PI) and distal intestine (DI) obtained from CTRL (empty bars) and Hi10-fed (slashed bars) (N = 5 fish/group). Data are reported as mean ± SEM with N = 5 fish/group. (**A**, **B**) statistical significance: ****P < 0.0001 versus CTRL DI; ^####^*P* < 0.0001; ^###^*P* < 0,001, ^##^*P* < 0.01 versus CTRL PI by Two-way ANOVA.

To evaluate the influence of *Hermetia illucens* meal on the excitatory neuromuscular response, non- cumulative concentration–response curves to the non-selective cholinergic agonist carbachol (CCh), were performed on longitudinally oriented proximal and distal intestine segments from all experimental groups. In the proximal intestine of the CTRL group, the concentration–response curve to CCh was lower than that obtained in the distal intestine of CTRL animals, with a significant decrease in maximum response (Emax) (*P* < 0.0001) (Fig. 4, panel A). *Hermetia illucens* meal did not influence concentration–response curves to CCh in the proximal intestine as compared to CTRL fishmeal, whereas in the distal intestine, the insect meal induced a significant downward shift of the CCh concentration–response curve, with a significant reduction in Emax as compared to CTRL conditions (*P* < 0.0001) (Fig. 4, panel A and B). Under all experimental conditions, EC_50_ values of the concentration–response curves did not differ significantly [CTRL proximal intestine: 0.17 (0.05–0.52) µM, n = 6; Hi10 proximal intestine: 0.20 (0.02–0.68) µM, n = 6; CTRL distal intestine: 0.14 (0.08–0.24) µM, n = 6; and Hi10 distal intestine: 0.13 (0.06–0.28) µM, n = 6]. To further investigate potential changes in the excitatory contractile function induced by *Hermetia illucens* meal, the effect of EFS was evaluated at increasing frequencies of stimulation on the proximal and distal longitudinal muscle. In the proximal intestine of CTRL animals, EFS-induced contractions were significantly lower than those observed in the distal intestine of CTRL animals (*P* < 0.01; Fig. 4, panel C). In the proximal intestine, *Hermetia illucens* meal induced a significant downward shift of the EFS-evoked contractile responses as compared to CTRL fishmeal (*P* < 0.001; Fig. 4, panel C). In the distal intestine, the insect meal did not significantly influence the EFS-induced contractile response with respect to CTRL fishmeal (*P* > 0.05; Fig. 4, panel C). In all groups EFS-mediated responses were of neuronal origin since they were totally abolished by neuronal blocker TTX (1 µM).

Cholineacetyltransferase (ChAT) antiserum stained the soma of large-, medium- and small-size myenteric neurons and fibers running along the longitudinal smooth muscle layer (Fig. 4, panel D). Myenteric neurons staining for ChAT displayed a variety of shapes: in the smaller-medium range they were usually oval, smooth and uniaxonal, while those in the large-medium range were uniaxonal and displayed an irregular profile with broad lamellar dendrites (Fig. 4, panel D). In the distal intestine of CTRL animals, the percentage of ChAT-positive myenteric neurons was slightly, but not significantly higher, than the value obtained in the proximal intestine (Fig. 4, panel E). After feeding with *Hermetia illucens* meal, the number of ChAT-positive myenteric neurons significantly increased (*P* < 0.0001) with respect to values obtained in the proximal intestine of CTRL animals, whereas in the distal intestine of Hi10 and CTRL animals, the number of ChAT-immunoreactive myenteric neurons was similar (Fig. 4, panel E).

### Changes in NANC excitatory neurotransmission in proximal and distal gilthead sea bream intestine after feeding with *Hermetia illucens* meal

**Figure 5:**
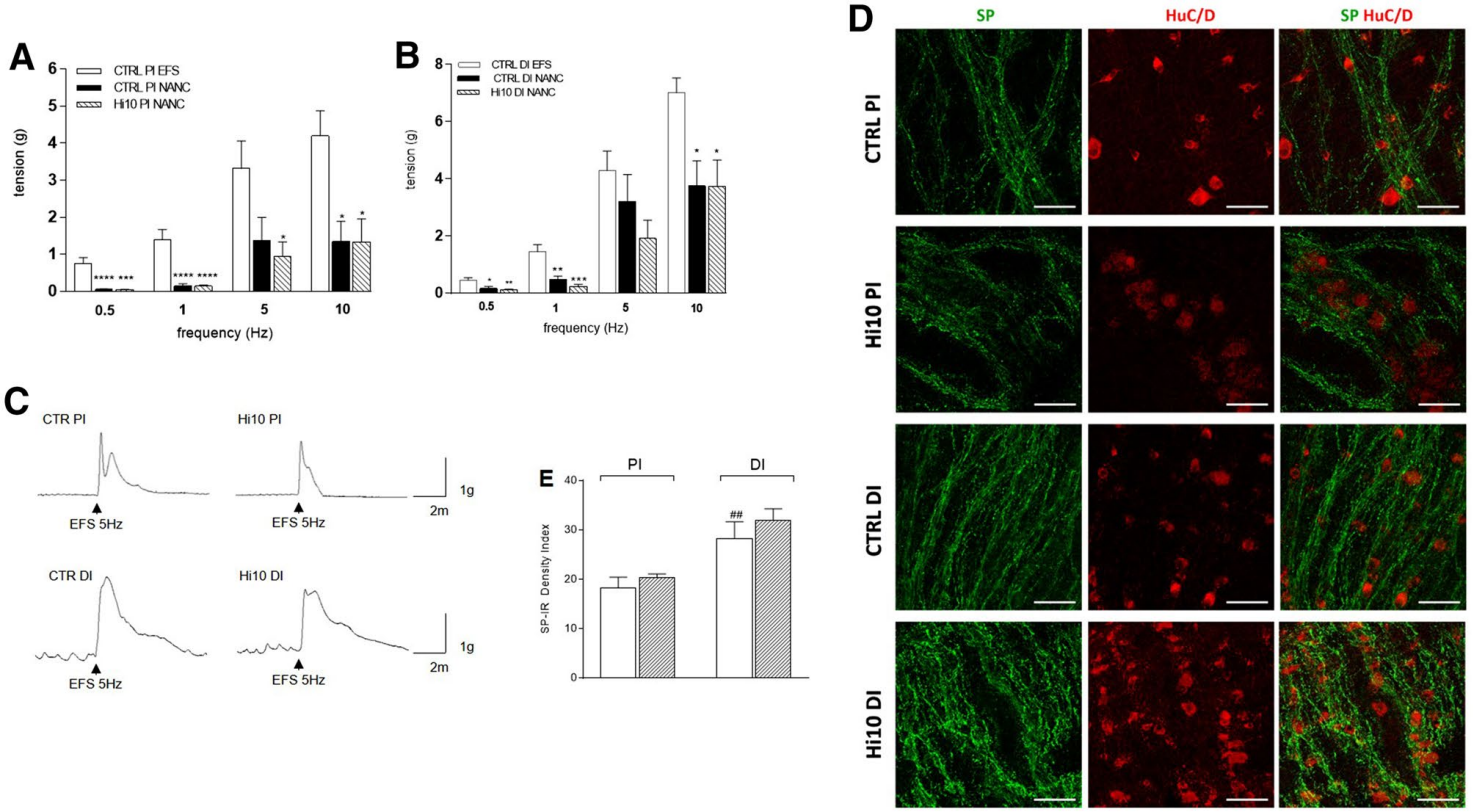
NANC contractions of both proximal (PI) and distal (DI) gilthead sea bream in CTRL and Hi10 fish. Contractile effect of increasing EFS frequencies in in the presence of atropine (1 µM) and guanethidine (1 µM) in the proximal (**A**) and distal intestine (**B**) of fishmeal diet (CTRL) and insect diet (Hi10) fed animals. Data obtained in NANC conditions are compared to EFS-evoked contractions in non-NANC conditions and are reported as mean ± SEM, N = 5 fish/group. Difference significance: ****P < 0.0001, ***P < 0.001, **P < 0.01, *P < 0.05 vs respective CTRL frequency of stimulation in non-NANC conditions, by one-way ANOVA with Sidak’s post-hoc test. (**C**) Representative tracings of 5 Hz EFS-evoked intestinal contractions in NANC conditions in the different experimental groups (**D**) Representative confocal microphotographs showing the distribution of SP (green) and HuC/D (red) in gilthead sea bream myenteric plexus in the different experimental groups (N = 5 fish/group). Scale bars = 50 μm. (**E**) Density index of SP immunoreactivity in proximal and distal intestine whole-mount preparations obtained in the different experimental groups (N = 5 fish/group). Data are reported as mean ± SEM, N = 5 fish/group. Difference significance: ^##^*P* < 0.01 versus CTRL PI, by one-way ANOVA with Sidak’s post-hoc test.

To assess the contribution of non-cholinergic neurotransmitters to proximal and distal intestinal motor function, EFS responses were evaluated under non-adrenergic non-cholinergic (NANC) conditions in the presence of atropine and guanethidine. In contrast to relaxation responses obtained under NANC conditions after EFS stimulation in most mammalian gut preparations, both in the proximal and distal intestine EFS stimulation under NANC conditions induced only frequency-dependent contractions (Fig. 5, panel A–C). In both gut regions, EFS-induced NANC contractions in the range of frequency stimulation between 0.5 and 1 Hz were significantly lower than EFS-induced contractions observed under non-NANC conditions, and this difference was particularly remarkable in the proximal intestine (*P* < 0.0001; *P* < 0.01; *P* < 0.05) (Fig. 5, panels A and B). EFS-induced NANC contractions at higher frequencies of 5–10 Hz were still lower, although less significantly (*P* < 0.05), than EFS-induced contractions under non-NANC conditions. At all frequencies, the fraction of the NANC excitatory component was higher in the distal than in the proximal intestine. Both in the proximal and distal intestine, *Hermetia illucens* meal did not affect EFS-evoked NANC contractions (Fig. 5, panels A and B). Analogously to mammals, SP represents a NANC excitatory neurotransmitter in fish gut; therefore, we evaluated SP staining in whole-mount preparations of proximal and distal gilthead sea bream intestine after feeding with fishmeal and with meal containing *Hermetia illucens* 10%. Immunoreactivity to SP was generally faint in the soma of prevalently scattered small- or medium- sized myenteric neurons and more intense in trunks of varicose fibers along the longitudinal muscle; thus, to evaluate possible changes in tachykinergic transmission, we measured the density index of SP staining (Fig. 5, panels D and E). In the distal intestine of CTRL animals, the SP density index was significantly higher (*P* < 0.01) than in the proximal intestine (Fig. 5, panel E). After feeding with *Hermetia illucens* meal, the SP density index did not significantly change with respect to values obtained in CTRL animals for both regions.

**Figure 6:**
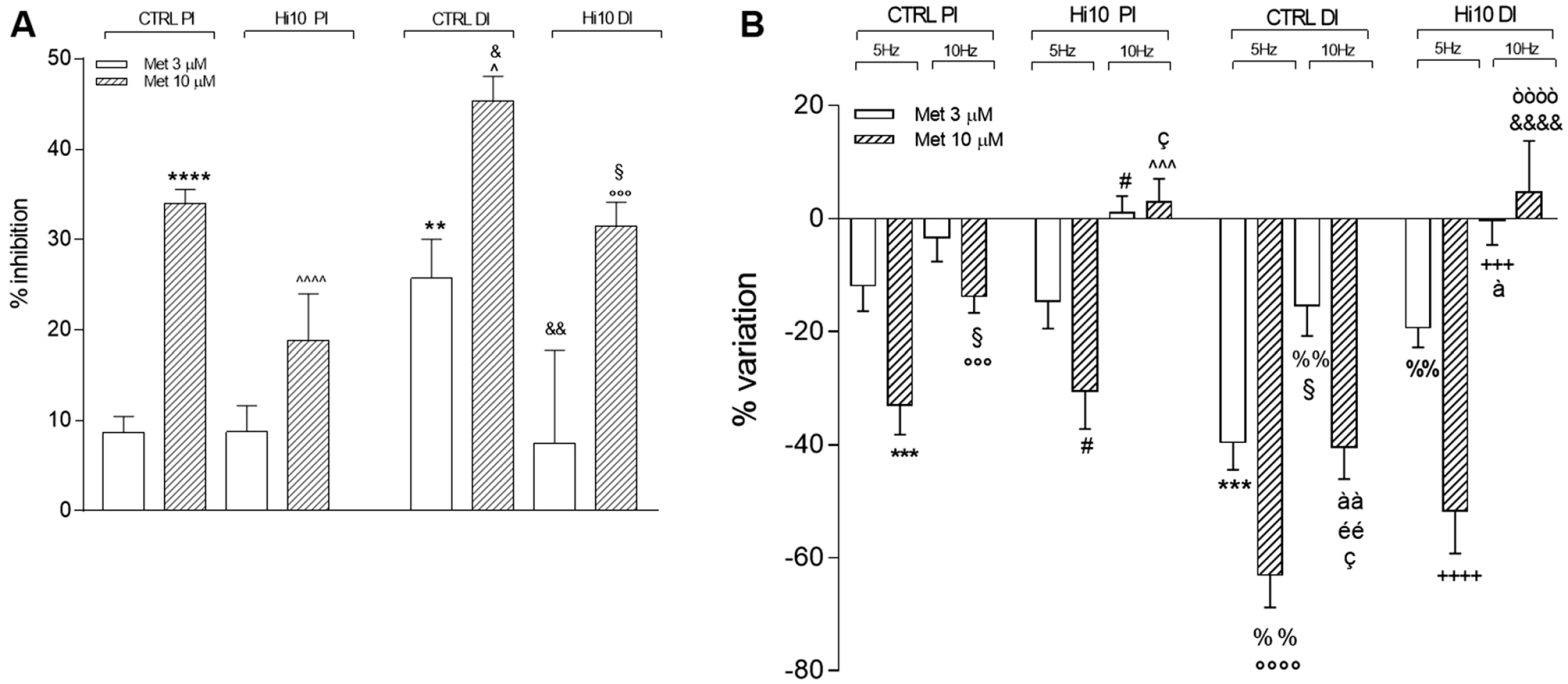
*Hermetia illucens* meal influences serotoninergic excitatory contractions in the proximal and distal intestine of the gilthead sea bream. (**A**) Inhibitory effect of the 5-HT antagonist, methysergide (3, empty bars, and 10 µM, dark upward diagonal bars) on spontaneous motor activity (**B**) and on NANC evoked contractions at 5 HZ and 10 Hz in the different experimental groups. Data are reported as mean ± SEM with N = 5 fish/group. (**A**) Statistical significance: ****P < 0.0001 and **P < 0.01 versus CTRL PI Met 3 µM; ^^^^*P* < 0.0001, ^*P* < 0.05 versus CTRL PI Met 10 µM; ^&&^*P* < 0.001 and ^&^*P* < 0.05 versus CTRL DI Met 3 µM; °°°*P* < 0.001 versus Hi10 PI Met 10 µM; ^§^*P* < 0.05 versus Hi10 DI Met 10 µM, by one-way ANOVA with Sidak’s post hoc test. (**B**) Statistical significance: ***P < 0.001 versus CTRL PI (5 Hz, Met 3 µM); °°°°*P* < 0.0001°°°*P* < 0.001 versus CTRL PI Met (5 Hz, 10 µM); ^§^*P* < 0.05 versus CTRL PI (10 Hz; Met 3 µM); ^ç^*P* < 0.05 versus CTRL PI (10 Hz Met 10 µM); ^#^*P* < 0.05 versus Hi10 PI (5 Hz; Met 3 µM); ^^^*P* < 0.001, ^*P* < 0.05 versus Hi10 PI (5 Hz Met 10 µM); ^%%^*P* < 0.01 versus CTRL DI (5 Hz, Met 3 µM); ^éé^*P* < 0.01 versus CTRL DI (5 Hz, Met 10 µM); ^à^*P* < 0.05 and ^ààà^*P* < 0.001 CTRL DI (10 Hz, Met 3 µM); ^òòòò^*P* < 0.0001 versus CTRL DI (10 Hz, Met 10 µM); ^&&&&^*P* < 0.0001 versus Hi10 DI (10 Hz, Met 10 µM);). ^+++^*P* < 0.001 AND ^++++^*P* < 0.0001 versus Hi10 DI (5 Hz; Met 3 µM), by one-way ANOVA with Sidak’s post hoc test.

**Figure 7:**
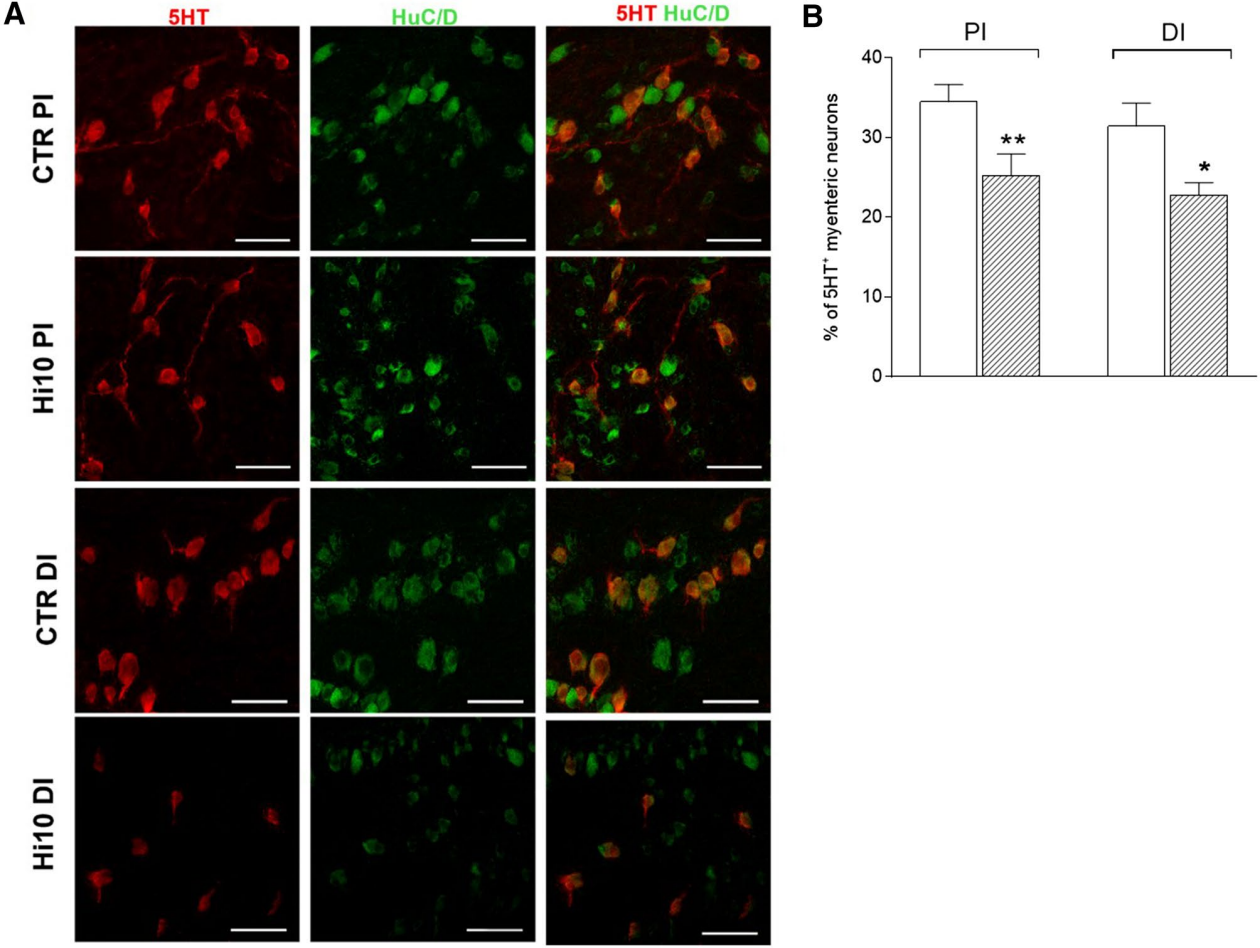
*Hermetia illucens* meal influences serotoninergic excitatory contractions in the proximal and distal intestine of the gilthead sea bream. (**A**) Immunohistochemical co-localization of 5-HT with the neuronal marker HuC/D in the different experimental groups. 5-HT immunoreactivity was detected in the soma and axon of myenteric neurons. Bars: 50 µm. (**B**) Percentage of 5-HT^+^ neurons in the proximal and distal sea bream intestine obtained from CTRL (empty bar) and Hi10 fed animals (dark upward diagonal bar). Data are reported as mean ± SEM, N = 5 fish/group. Difference significance: ^*^P < 0.05 versus CTRL DI, ^**^P < 0.01 versus CTRL PI by one-way ANOVA with Sidak’s post-hoc test.

5-HT is another important enteric neurotransmitter, which has been proposed to be an excitatory non-cholinergic transmitter to smooth muscle in fish^7,12^. Thus, we have evaluated the effect of the nonselective 5-HT receptor antagonist methysergide (3 and 10 µM) on 5 and 10 Hz EFS-induced contractions under NANC conditions, which were more prominently induced by a non-cholinergic component. Both in the proximal and distal intestine of CTRL and Hi10 animals, methysergide reduced spontaneous motility in a concentration-dependently manner (Fig. 6, panel A). In the CTRL group, both 3 and 10 µM methysergide induced a significantly higher inhibitory effect on the distal intestine than on the proximal intestine (*P* < 0.01 and *P* < 0.05, respectively); in the Hi10 group, a higher inhibitory effect of methysergide in the distal than in the proximal intestine could only be demonstrated at the concentration of 10 µM. In proximal intestine of Hi10 animals, the 10 µM methysergide-induced inhibitory effect was lower than the effect observed in the CTRL group at the same drug concentration (*P* < 0.0001), while in the distal intestine of Hi10 animals, methysergide-mediated inhibition was lower at both concentrations tested (*P* < 0.01 and *P* < 0.05) (Fig. 6, panel A). In both proximal and distal intestinal segments obtained from CTRL animals, methysergide induced a concentration-dependent inhibitory effect on NANC EFS contractions induced at both 5 and 10 Hz (Fig. 6, panel B). In both intestinal regions, the inhibitory effect of methysergide was lower at 10 Hz than at 5 Hz, reaching values significantly different from those obtained at 5 Hz for 10 µM methysergide in the proximal intestine (*P* < 0.001) and for both 3 and 10 µM methysergide in the distal intestine (*P* < 0.01). In the proximal and distal intestine of *Hermetia illucens* meal-fed animals, the serotoninergic antagonist induced a concentration-dependent inhibitory effect on NANC EFS-induced contractions at 5 Hz. However, in this experimental group, the inhibitory effect of methysergide was totally abolished at 10 Hz and reverted to a contractile response at 10 µM (Fig. 6, panel B). In the distal intestine of Hi10-fed animals, the inhibitory effect of methysergide was significantly lower than in the distal intestine of the CTRL group, reaching statistically significant values only at 3 µM (*P* < 0.01). In the Hi10 group, the inhibitory effect of 3 µM methysergide at 10 Hz EFS in NANC conditions was significantly lower than in the distal intestine of the CTRL group (*P* < 0.001) and changed into a contractile response with 10 µM methysergide (Fig. 6, panel B). In the distal intestine of CTRL animals, both methysergide concentrations had significantly higher inhibitory effects on NANC contractions both at 5 and 10 Hz than those observed in the proximal intestine (PI 5 Hz: *P* < 0.01 and *P* < 0.001, respectively, for 3 µM and 10 µM methysergide; 10 Hz: *P* < 0.05 and *P* < 0.001, respectively, for 3 µM and 10 µM methysergide). 5-HT immunoreactivity was predominantly found in the soma of either single neurons or neurons forming groups of 2–4 cells and in axonal prolongations (Fig. 7, panel A). The percentage of 5-HT-positive myenteric neurons as determined by co-staining with HuC/D was similar in the proximal and distal intestine of CTRL animals (Fig. 7, panel B). After feeding with *Hermetia illucens* meal, the percentage of 5HT^+^ neurons was significantly lower in both the proximal and distal intestine (*P* < 0.01 and *P* < 0.05, respectively) (Fig. 7, panel B).

### Influence of *Hermetia illucens* meal on the inhibitory relaxation response of the proximal and distal intestine in gilthead sea bream

**Figure 8:**
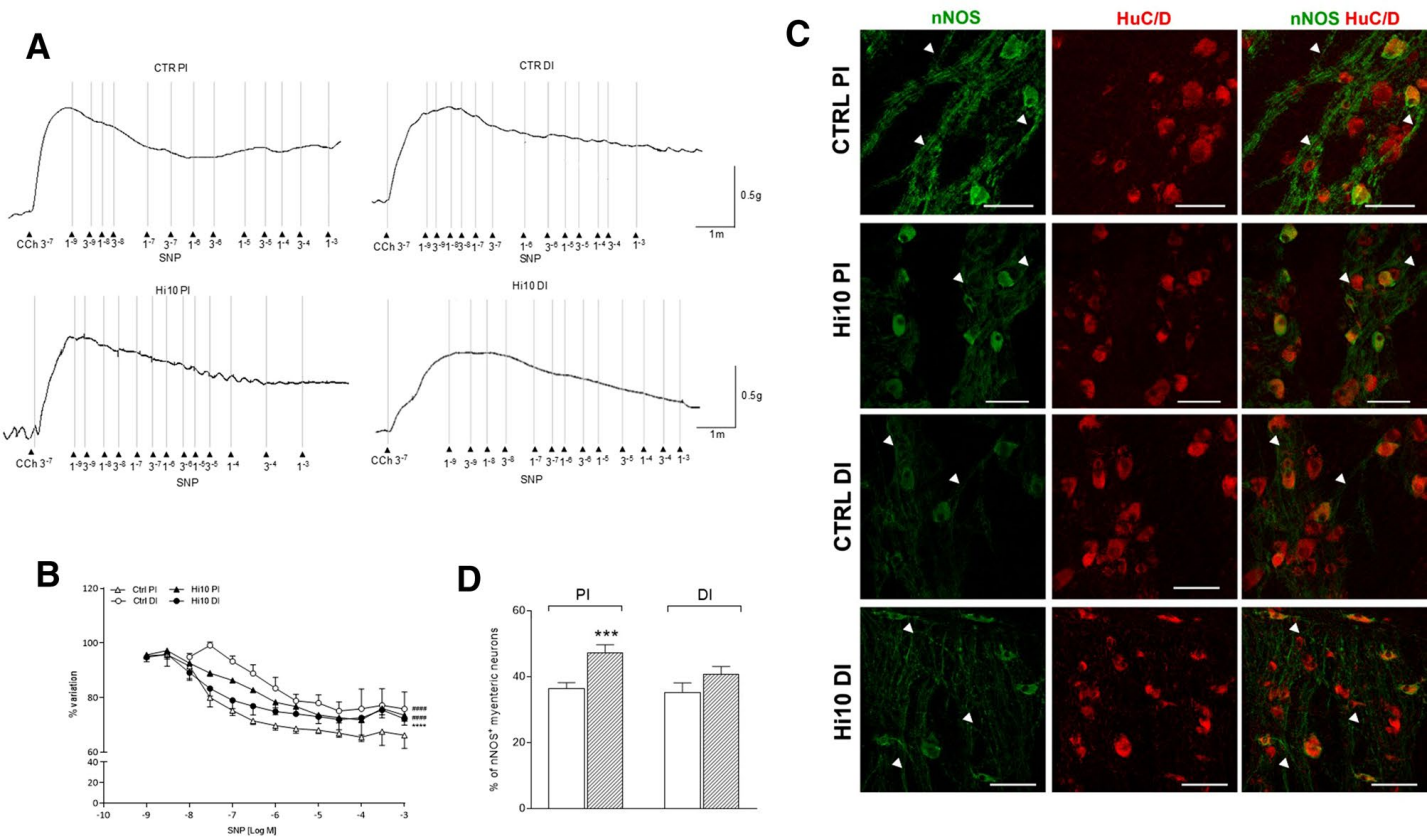
Effect of the nitric oxide donor, sodium nitroprusside (SNP) on the proximal and distal gilthead sea bream obtained in the different experimental conditions. (**A**) Tracings of relaxation concentration response curves to SNP (from 1 nM to 1 mM) of CCh (0.5 µM) precontracted segments obtained from the proximal and distal intestine. (**B**) Effect of SNP on CCh-induced contractions (N = 5 fish/ group). Data are reported as mean ± SEM. Statistical significance: ^####^*P* < 0.001 versus CTRL PI; ****P < 0.0001 versus CTRL DI by two-way ANOVA (**C**) Representative confocal photomicrographs showing the distribution of nNOS immunoreactive myenteric neurons (red) and their co-localization with pan neuronal marker HuC/D (green). Bars: 50 μm.(**D**) Percentage of nNOS^+^ neurons in the proximal and distal sea bream intestine obtained from CTRL (empty bar) and Hi10-fed animals (slashed bar). Data are reported as mean ± SEM, N = 5 fish/group. Difference significance: ^***^P < 0.001 versus CTRL PI, by one-way ANOVA with Sidak’s post-hoc test.

The nitric oxide donor sodium nitroprusside (SNP) reduced the amplitude of the spontaneous contractions of the longitudinal muscle in both the proximal and distal intestine of both CTRL and Hi10 animals in a concentration-dependent manner from 1 to 100 µM (data not shown). In all experimental groups, spontaneous contractions were similarly reduced down to 70% of the initial value after adding 100 µM SNP. However, in CCh-contracted proximal and distal gilthead sea bream intestine, the inhibitory effect of SNP was dependent on region and diet. In both the CTRL and Hi10 groups, concentration–response curves to SNP were shallow and preparations did not relax towards basal tone values even at a concentration of 1 mM, the highest concentration tested (Fig. 8, panel A and B). In CTRL animals, the SNP concentration–response curve obtained in the distal intestine was shifted to the right with respect the curve obtained in the proximal intestine (*P* < 0.0001). This shift was confirmed by the significant difference in EC_50_ values obtained by unpaired Student’s t test analysis in the two regions [(CTRL PI EC_50_: 28.2 (1.82–68.9) nM and CTRL DI: 0.41 (0.02–1.52) µM; *P* < 0.05)]. After feeding with *Hermetia illucens* meal, in the proximal intestine, the inhibitory effect of SNP was lower than the effect obtained from CTRL animals as indicated by significantly different concentration–response curves (*P* < 0.0001); however, the EC_50_ value was not significantly different from that obtained in CTRL animals [(Hi10 PI EC50: 98.0 (1.81–336) nM] (Fig. 8, panel B). After feeding with *Hermetia illucens* meal, in the distal intestine, the concentration–response curve to SNP was significantly shifted to the left with respect to the curve obtained from CTRL animals (*P* < 0.0001), displaying a significantly different EC_50_ value [(Hi10 DI EC50: 20,1 (0.14–59.3) nM; *P* < 0.01, by unpaired Student’s t test] (Fig. 8, panel B).

In LMMP whole-mount preparations of both proximal and distal intestine from both experimental groups, nNOS-IR was observed in the soma of small to large neurons and in nerve fibres running along the longitudinal smooth muscle layer (Fig. 8, panel C).

The percentage of nNOS-positive myenteric neurons, determined by co-staining with HuC/D, in the proximal intestine of CTRL animals was not significantly different than the value obtained in the distal intestine (Fig. 8, panel D**)**. After feeding with *Hermetia illucens* meal, the number of nNOS^+^ myenteric neurons in the proximal intestine was significantly higher than in CTRL animals (*P* < 0.001). In the distal intestine of Hi10-fed fish, the number of nNOS^+^ myenteric neurons showed a slight, but not significant, increase with respect to CTRL values (Fig. 8, panel D).

## Discussion

This study describes the consequences of partially substituting fishmeal with 10% *Hermetia illucens* (Hi10) larvae meal on gastrointestinal motor function in adult gilthead sea bream. In particular, we analyzed possible diet-related changes in myenteric plexus chemical coding, in neuromuscular responses in both proximal and distal intestine in vitro, and in gastrointestinal transit in vivo*.* In both intestinal regions, we showed that the addition of insect meal to conventional fishmeal induces adaptive changes in some myenteric neuron populations as well as changes in the excitatory and inhibitory components of peristalsis, reducing the efficiency of gastrointestinal transit.

A gross morphological evaluation of intestinal specimens obtained from the proximal and distal intestine did not show any major differences between fishmeal- and Hi10-fed fish, suggesting that changes in diet composition did not significantly affect the gross anatomy of gilthead sea bream intestine. In addition, Hi10 diet was not associated with significant remodelling of the mucosal layer, which represents a response to stressor or inflammatory insult after fish are exposed to alternative feedstuffs such as soybean meal^13,14^. However, a more detailed histochemical evaluation of the intestinal wall demonstrated reduced villi density and width in the epithelial layer of the proximal intestine. Although these changes may influence digestion by reducing nutrient absorption capacity^13,15^, we did not observe any significant alterations in weight and growth of the Hi10-fed fish as compared to CTRL animals. Furthermore, the morphology of the distal intestine epithelial layer, which in teleost fish is partially responsible for nutrient digestion and absorption, remained unaltered^16^. The absence of an overt inflammatory injury after feeding with Hi10 meal is also suggested by the unchanged submucosal layer architecture and thickness, both in the proximal and distal intestine. However, a low-grade inflammatory response cannot be excluded, as suggested by the increased *muscularis propria* thickness, which was particularly prominent in the distal intestine of Hi10-fed fish^17^. In accordance with previous studies^8^, myenteric neurons were unevenly distributed over the longitudinal muscle in the neuromuscular compartment of both proximal and distal intestine of CTRL and Hi10 fish, as revealed by staining with the pan neuronal marker HuC/D. HuC/D immunoreactivity was uniformly distributed in the cytosol of myenteric neurons and did not display nuclear translocation, which occurs under different pathological conditions, including inflammation injury^18^. The unaltered viability of myenteric neurons after feeding with Hi10 meal was also confirmed by the unchanged density of myenteric neurons both in the proximal and distal intestine.

To investigate whether adding *Hermetia illucens* larva meal to the gilthead sea bream diet might modulate the intestinal neuromuscular function, we evaluated the efficiency of gastrointestinal transit in vivo. Indeed, the significant reduction in the geometric center of fluorescence distribution in Hi10-fed animals suggests that changes occurred in ENS circuitries, modulating the ascending contraction or descending inhibition, or both, during the peristaltic reflex. Therefore, we investigated both components by performing an in vitro organ bath assay and by immunohistochemically evaluating the distribution of the relevant neurotransmitter pathways. In teleost fish, as in mammals, acetylcholine (ACh) represents the major neurotransmitter released from excitatory motor neurons to stimulate the excitatory ascending smooth muscle contraction^7,12^. In CTRL animals, around 20% of myenteric neurons stained for the cholinergic marker cholineacetyltransferase (ChAT) both in the proximal and distal intestine, which is in good agreement with previously obtained data in juvenile gilthead sea bream intestine^8^. In CTRL animals, the contractile response to the muscarinic agonist carbachol (CCh) was more prominent in the distal than in the proximal intestine, as suggested by the significantly higher E_max_ of the dose–response curve, indicative of higher postjunctional cholinergic responses in the distal than in the proximal intestine of adult gilthead sea bream. Furthermore, cholinergic postjunctional responses were more sensitive to Hi10 meal in the distal intestine than in the proximal intestine as, after feeding with insect meal, maximal contractions to CCh were significantly reduced in the distal intestine and only slightly diminished in the proximal region. This reduction was not associated with a decreased number of ChAT-positive neurons, further strengthening the concept that, in the distal intestine, Hi10 diet modulates the muscarinic postjunctional component of the cholinergic response. In CTRL animals, neuronally-mediated tetrodotoxin-sensitive EFS-induced contractions were also significantly higher in the distal intestine than in the proximal intestine. However, in contrast to what was observed for CCh-induced contractions, EFS-induced contractions were significantly lower in the proximal but not in the distal intestine after feeding with Hi10 meal. In the myenteric plexus of the proximal, but not distal intestine of Hi10-fed animals, the number of ChAT^+^ neurons was significantly upregulated, possibly representing a positive homeostatic feedback mechanism to compensate for reduced neuronal responses. Interestingly, the sensitivity of cholinergic pathways to alimentary changes has also been described in rodents^19,20^. In adult gilthead sea bream ENS, other excitatory neurotransmitters, such as SP and 5HT, may be involved in excitatory contractile responses^8,21^. In teleost, SP has been described as a co-transmitter in cholinergic excitatory neurons to the smooth muscle layers^12,21^. In this study, the non-cholinergic component of the excitatory response was evaluated after EFS stimulation under nonadrenergic noncholinergic (NANC) conditions, i.e., in the presence of atropine and guanethidine, to block cholinergic and adrenergic responses. Under these conditions, the mammalian intestine generally responds with an on-relaxation followed by a rebound contraction, which is mainly tachykinergic. However, in agreement with other studies, transmural stimulation under NANC conditions was excitatory inducing only a contraction in both proximal and distal segments of the gilthead sea bream intestine^22^. Notably, both in the proximal and distal intestine of both CTRL and Hi10 animals, EFS-induced contractile responses at 0.5 and 1 Hz were almost completely abolished after administering atropine, suggesting a major involvement of the cholinergic component at low frequencies, as expected^23,24^. Conversely, in both CTRL and Hi10-fed animals, EFS-induced contractile responses at 5 and 10 Hz were less sensitive to atropine suggesting that non-cholinergic neurotransmitters, such as SP, 5HT, or ATP are involved both in the proximal and distal intestine^8,22,25^. At all frequencies, the NANC excitatory component was higher in the distal than in the proximal intestine of both CTRL and Hi10 animals. This observation correlates well with the significantly higher SP density index staining in whole-mount preparations obtained from the distal than from the proximal intestine in both CTRL and Hi10-fed gilthead sea bream.

Serotonin (5-HT) has also been reported to play a key role in modulating enteric reflexes. In the mammalian intestine, the main source of 5-HT is represented by enterochromaffin (EC) cells, whereas in fish intestine most 5-HT is stored in enteric nerves^26^, acting as an excitatory non-cholinergic transmitter to smooth muscle^23,27^ and as a modulator of both ascending and descending interneuronal pathways^28^ In both proximal and distal intestine of CTRL animals, the 35% percent of myenteric neurons were immunoreactive to 5HT, reflecting the high density level of serotoninergic neurons observed in other adult teleost fish such as rainbow trout (*Oncorhynchus mykiss*), sand flathead (*Platycephalus bassensis*), smooth toadfish (*Tetractenos glaber*) and short-finned eel (*Anguilla australis*) with respect to mammals^26,29^. In analogy to data obtained in the rainbow trout intestine^27^, the ability of the non-selective 5HT receptor antagonist methysergide to inhibit spontaneous motility as well as EFS-induced contractions at 5 and 10 Hz both in the proximal and distal intestine of CTRL animals in a concentration-dependent manner in the present study, suggests that the amine plays a physiologically relevant excitatory role for fish gut motility. These observations further underscore the differences between the mammalian and fish gut serotoninergic system as depletion of endogenous 5HT from guinea-pig and mice gut neither blocked peristalsis nor reduced transit in vivo^30,31^. Interestingly, the distal gilthead sea bream intestine was more sensitive to methysergide than the proximal intestine, suggesting that the excitatory serotoninergic response is more pronounced in the distal than in the proximal intestine, as observed for the excitatory cholinergic component. During EFS stimulation both in the proximal and distal intestine, the inhibitory effect of methysergide was reduced with increasing frequencies of stimulation. Such loss of function may be caused when serotoninergic interneurons impinging on inhibitory descending motor neurons are activated or, alternatively when 5HT release or the release of other neurotransmitters from excitatory motor neurons is reduced at higher frequencies^12,32^. After feeding with Hi10 meal, the inhibitory effect of methysergide both on spontaneous motility and on EFS-induced contractions was significantly reduced, especially in the distal intestine. More importantly, methysergide-mediated inhibition at 10 Hz EFS was absent in both regions, suggesting that the insect meal may negatively affect the excitatory serotoninergic pathways. The reduced effect of methysergide well matches with the reduced density of 5HT^+^ myenteric neurons in both proximal and distal intestine of Hi10 fish.

Since in NANC conditions EFS did not induce any relaxation of both proximal and distal intestine, to study inhibitory relaxation responses in our model, we evaluated the effect of increasing concentrations of the nitric oxide (NO) donor, sodium nitroprusside (SNP). SNP concentration-dependently reduced both spontaneous and CCh-precontracted intestinal segments, as described in previous studies^33^. Relaxation responses to SNP in CCh-precontracted segments were dependent on region and diet. In CTRL animals, SNP displayed a higher potency in relaxing the proximal than the distal intestine, and this observation is in good agreement with the higher contractile capacity of the distal than the proximal intestine, reflecting different digestive functions of these two intestinal regions^16^. From a mechanistic view point, the different degree of SNP-mediated relaxation may depend upon a diverse modulation of nitric oxide-activated signal transduction pathways, namely cGMP signalling, in smooth muscle cells^34^. Alterations of neuronal nitrergic inhibitory pathways seem to be excluded as no differences were detected in the density of nNOS^+^ neurons between these segments. Both in the proximal and distal intestine, SNP displayed a limited efficacy in relaxing the CCh-precontracted longitudinal smooth muscle as, at the highest concentration tested, the maximal inhibition was around the 35% of the initial tension. We cannot exclude that other inhibitory neurotransmitters, such as VIP, whose presence has been demonstrated in juvenile gilthead sea bream and the myenteric plexus of other teleost fish, may contribute to the descending relaxation in adult gilthead sea bream, too^8,35^. After feeding with Hi10 meal, the inhibition-response curve to SNP was significantly shifted to the right in the proximal intestine, indicating a reduced relaxation response. In contrast, the dose–response curve to SNP was shifted to the left in the distal intestine of Hi10 fish, indicating an increase in the relaxation response under this dietary condition. An increase in the number of nNOS^+^ neurons in the myenteric plexus in the proximal intestine may represent a homeostatic positive feedback to compensate for the reduced inhibitory postjunctional response.

## Conclusions

This study has shown for the first time that partially substituting fishmeal with insect meal in the diet of gilthead sea bream may induce changes in the gastrointestinal transit time, in the efficiency of the excitatory and inhibitory components of the peristaltic reflex, and in the chemical coding of some major myenteric neurotransmitters, such as cholinergic, serotoninergic and nitrergic pathways. The mechanism/s underlying such changes need to be elucidated. We cannot exclude that some components of insect meal may influence myenteric plexus circuitries either directly or indirectly by influencing other cellular components of the enteric microenvironment, such as immune cells by activating neuroimmune responses, or via the gut microbiota within the lumen^36^. The observed alterations did not, however, have unfavorable consequences for animal growth, suggesting that adding 10% Hi meal may represent a good alternative to fishmeal protein source in gilthead sea bream diets. However, in view of the observed changes in gastrointestinal motor function and myenteric plexus chemical coding, future studies are needed to determine the effects of longer feeding periods with the same or even higher inclusion levels of *Hermetia illucens* larvae meal on marine fish growth performance.

## Materials and methods

### Animals and tissue sampling

Experiments were conducted at the aquaculture laboratory of the University of Insubria. One hundred sixty eight gilthead sea bream (*Sparus aurata*), (mean initial weight 233.06 ± 5.52 and length 22.20 ± 0.17), purchased from Società Agricola CIVITA ITTICA S.r.l. (Verona, Italy), were randomly distributed into 6 circular fiberglass tanks of 700 L (28 fish/tank; initial density < 10 kg/m^3^) at a temperature of 19 ± 1.5 °C and pH 8.3 ± 0.4. Animals were continuously monitored during the trial, and dissolved oxygen concentration (maintained over 85%), total ammonia nitrogen, and salinity were periodically measured. After 10 days of acclimation, fish were fed ad libitum once per day for 96 days with two experimental diets in triplicate (3 tanks/diet). A group of control fish (CTRL), received a standard diet containing 20% fishmeal as well as other protein sources (wheat gluten, soybean meal, and hemoglobin), whereas another group of animals (Hi10) received a diet containing 10% of *Hermetia illucens* (Hi) larva meal as a partial replacement of the fishmeal contained in the CTRL diet (Supplementary Table S3). This percentage of *Hermetia illucens* larva meal represents a cost effective and tolerable meal substitution^37,38^. The Hi larva meal was provided by HiProMine S.A. (Robakowo, Poland). At the end of the feeding trial 30 gilthead sea bream obtained from either the CTRL group and Hi10 group were rapidly anesthetized with tricaine methansulfonate (MS222, 300 ppm) and then euthanized by severing the spinal cord. The whole intestine was rapidly dissected out, and the proximal intestine was separated from the distal intestine. Intestinal segments were then rinsed with an ice-cold Krebs solution [composition (mM): 118 NaCl, 4.8 KCl, 2.5 CaCl_2_∙2H_2_O, 1.2 MgCl_2_ 6H_2_O, 1.25 NaH_2_PO_4_, 25 NaHCO_3_, and 11 Glucose]. Whole-wall intestinal segments were either fixed and stored for subsequent immunohistochemistry experiments or used for organ bath experiments. Animal care and handling was in accordance with ARRIVE guidelines and accomplished the provisions of the European Union Council Directive 2010/63, recognized and adopted by the Italian Government (DLgs No. 26/2014). The protocol was approved by the Animal Care and Use Ethics Committee of the University of Insubria and by the Italian Ministry of Health (n°285/2020-PR).

### Histology

For histological evaluation, proximal and distal intestinal segments of 6 fish/diet were collected, dehydrated, and embedded in paraffin according to routine techniques^8^. Intestinal samples were successively cut into 5 μm cross sections, and stained with hematoxylin–eosin (H&E) for light microscope analysis. The histological assessment was performed measuring 7 different crucial morphological criteria as described by Escaffre et al., 2015^39^, consisting in villi height, width and density of submucosal layer thickness, total smooth muscle thickness, circular smooth muscle thickness and longitudinal smooth muscle thickness. Images were obtained with an Olympus IX51 optical microscope and using Fiji software (open-source Java-based imaging program). Villi density was evaluated in five cross sections for each intestinal region for each fish, whereas 10 measurements of each of the other parameters were performed both in the proximal and distal gut portion for each fish.

### Whole-mount immunohistochemistry

Segments of the proximal and distal gilthead sea bream intestine were fixed according to Giaroni et al. 2009^40^ with 0.2 M sodium phosphate-buffer (PBS composition [M]: 0.14 NaCl, 0.003 KCl, 0.015 Na2HPO4, 0.0015 KH2PO4, pH 7.4) containing 4% formaldehyde plus 0.2% picric acid for 2 h at room temperature (RT). Preparations were then cleared of fixative and stored at 4 °C in PBS containing 0.05% thimerosal. Longitudinal muscle myenteric plexus (LMMP) whole-mount preparations were prepared according to the method described by Ceccotti et al.^8^. LMMPs were then exposed to a PBS solution containing 1% Triton X-100 and 10% normal horse serum (NHS) for 1 h at RT (Euroclone, Celbio, Milan, Italy), to permeabilize the tissue and to block non-specific binding sites. Successively, tissues were incubated with optimally diluted primary antibodies. Double-labeling was performed during consecutive incubation times of the primary and secondary antibodies, whose optimal dilutions are described in Supplementary Table S4^40^. Preparations were mounted onto glass slides, using a mounting medium with DAPI (Vectashield®; Vector Lab, Burlingame, CA, USA). Neuron counts were made on HuC/D stained LMMPs, obtained from five animals/diet, digitized by capturing as many as 40 × objective microscope fields (0.1406 mm^2^) as possible (4–5 fields/fish for a total of 5 fish/group). The neuron count obtained for each field was divided by the total image field area and expressed as the number of neurons/mm^2^^41^.

To establish the proportion of neuronal nitric oxide synthases (nNOS), serotonin (5-HT), choline acetyltransferase (ChAT) and substance P (SP) expressing myenteric neurons, quantitative analysis of double fluorescently labelled small intestine whole mounts was performed as previously described^42^. A total of 5 fields were sampled from LMMP preparations obtained from the proximal and distal intestine of five animals/diet. Immunoreactivity for SP was assessed by analyzing the density index of labelling per intestinal surface area (10 fields per preparation at 40 × magnification)^42^. Negative controls and interference control staining were evaluated by omitting both primary and secondary antibody, and by incubating colonic whole-mounts with non-immune serum from the same species in which the primary antibodies were raised. In all these conditions, no specific signal was detected. Preparations were analyzed using a Leica TCS SP5 confocal laser scanning system (Leica Microsystems GmbH, Wetzlar, Germany) and pictures were processed with Adobe-Photoshop CS6S software.

### Gastrointestinal transit analysis

Gastrointestinal transit was measured by evaluating the distribution of an intragastric gavage of fluorescein isothiocyanate (FITC)-labeled dextran (250 kDa, FD250; 6.25 mg/mL dissolved in 0.9% saline) as described by Bistoletti et al., 2020, with modifications^42^. Gilthead sea bream from CTRL and Hi10 groups were given the FITC dextran gavage at 9 am and euthanized 6 h after FD250 kDa administration and the entire GI tract was carefully removed and divided into 11 segments: a single stomach segment (sto), a single pyloric ceca segment, 9 equal-length segments of proximal intestine (S1–S3), median intestine (S4–S6), distal intestine (S7–S8) and a single segment for the rectum. Luminal contents from each sample were collected and clarified by centrifugation (12,000 rpm, 10 min). The cleared supernatants were fluorimetrically measured in duplicate for FD250 intensity, at 494/521 nm using a microplate reader (Infinite 200pro, TECAN). Data were expressed as the percentage of fluorescence for each segment with respect to the total fluorescence along the gastrointestinal tract. The efficiency of FITC-dextran transit along the gastrointestinal tract was determined by calculating the geometric center (GC) for the distribution of the fluorescent probe using the following equation: GC = Σ (% of fluorescence signal per segment x segment number)/100^42^.

### Excitatory and inhibitory in vitro motor responses

The effects of *Hermetia illucens* meal on gilthead sea bream intestinal neuromuscular function were examined in vitro by measuring the excitatory contractile response of the longitudinal muscle, which triggers the preparative phase of the peristaltic reflex and is synchronous with circular muscle contraction during peristalsis, prompting propulsive bowel activity^43^. In vitro excitatory and inhibitory responses of both proximal and distal longitudinal muscle gilthead sea bream intestine were measured according to Bistoletti et al., 2020^42^. Briefly, segments of the small and distal intestine (1 cm) were rapidly excised, flushed with Krebs solution [composition (mM): 118 NaCl, 4.8 KCl, 2.5 CaCl_2_∙2H_2_O, 1.2 MgCl_2_∙6H_2_O, 1.25 NaH_2_PO_4_, 25 NaHCO_3_, and 11 Glucose], cleared of connective and fat tissue and mounted in isolated baths containing 10 mL of continuously oxygenated (95% O_2_ and 5% CO_2_) and heated (22 ± 1 °C) Krebs solution. Silk ligatures were applied to each end of the segment positioned along the longitudinal axis; one end was attached to a rigid support and the other to an isometric force displacement transducer (MDE Research GmbH, Walldorf, GE). Mechanical activity was recorded with a PowerLab acquisition data system 8 (AD Instruments, UK) and elaborated with a LabChart 4.0 program (AD Instruments, UK). An initial load of 1 g was applied to each intestinal specimen. Tissues were allowed to equilibrate for 60 min prior to the start of the experiments. The average amplitude of spontaneous contractions, expressed as g tension/tissue weight, and the frequency of phasic contractions, expressed as number of contractions/secs were determined 20 min before starting the experiment. For each segment, concentration–effect curves to the muscarinic agonist, carbachol (CCh) were constructed non-cumulatively to evaluate postjunctional cholinergic responses expressed as g tension/tissue weight. The effect of increasing doses of CCh was plotted into a nonlinear regression model (fitted to a sigmoidal equation) to calculate EC_50_ and maximal tension (E_max_) values. Neuronally mediated contractions, were obtained by Electric Field Stimulation (EFS, 0.5–20 Hz; 1-ms pulse duration, 10-s pulse train, 40 V) using platinum bipolar co-axial electrodes, attached to an MDE electronic stimulator (MDE Research) and were expressed as g tension/tissue weight. Frequency–response curves were repeated in the presence of tetrodotoxin (TTX; 1 µM) to identify if the response was of neuronal origin.

Non-adrenergic non-cholinergic (NANC) responses were measured at increasing frequencies (EFS, 0.5–10 Hz; 1-ms pulse duration, 10-s pulse train, 40 V), after allowing a 20 min incubation period with atropine (1 μM) and guanethidine (1 μM). Under NANC conditions, EFS induced contractions at 5 Hz and 10 Hz were evaluated also after 20 min incubation with the 5-Hydroxytryptamine (5-HT) antagonist, methysergide (3 µM and 10 µM).

In vitro intestinal relaxations were studied by evaluating the effect of the nitric oxide (NO) donor, sodium nitroprusside (SNP). To this end, relaxation concentration–response curves to SNP (1 nM–1 mM) were cumulatively constructed on CCh-precontracted (0.5 µM) segments. Relaxant responses, expressed as percentage reduction of the CCh-induced contraction, were plotted into a nonlinear regression analysis model (fitted to a sigmoidal equation) to calculate EC_50_ and maximal relaxation (E_max_) values.

### Statistical analysis

Data are expressed as mean ± standard error of the mean (SEM), except for the geometric center, which is presented as median and range (minimum–maximum), of at least 5 experiments. Statistical analysis of growth performance was performed considering the tank as an experimental unit. Statistical significance was calculated by unpaired Student’s t test, one-way ANOVA followed by Sidak’s post-hoc test or by two-way ANOVA for multiple variables, where appropriate using the GraphPad Prism software (GraphPad 7 Software Inc, La Jolla, USA). Differences were considered statistically significant when *P* values were < 0.05.

## Supplementary Information

Supplementary Information.

## Abbreviations

H&E: Hematoxylin-eosin
LMMP: Longitudinal muscle myenteric plexus
nNOS: Neuronal nitric oxide synthases
5-HT: Serotonin
ChAT: Cholineacetyltransferase
SP: Substance P
GC: Geometric center
CCh: Carbachol
EFS: Electric field stimulation
TTX: Tetrodotoxin
NANC: Non-adrenergic non-cholinergic
NO: Nitric oxide
SNP: Sodium nitroprusside
ENS: Enteric nervous system
ACh: Acetylcholine
FCR: Food conversion ratio
SGR: Specific growth rate
FTD: FITC-labelled dextran
PI: Proximal intestine
DI: Distal intestine
EC: Enterochromaffin cells
Hi: *Hermetia illucens*
NHS: Normal horse serum
VIP: Vasoactive intestinal peptide
