# Supplementary material for: Effect of partial substitution of fishmeal with insect meal (Hermetia illucens) on gut neuromuscular function in Gilthead sea bream (Sparus aurata)
Source: Sci Rep. 2021 Nov 8;11:21788. doi: 10.1038/s41598-021-01242-1 (PMC8575790; doi:10.1038/s41598-021-01242-1)
Supplement: Supplementary file 1 — Supplementary Information. [file 41598_2021_1242_MOESM1_ESM.pdf]

Effect of partial substitution of fishmeal with insect meal (*Hermetia illucens*) on gut neuromuscular function in Gilthead sea bream (*Sparus aurata*)

Annalisa Bosi[1], Davide Banfi[1], Federico Moroni[2], Chiara Ceccotti[2], Maria Cecilia Giron[3], Micaela Antonini[2], Cristina Giaroni[1]*, Genciana Terova[2]

[1]Department of Medicine and Surgery, University of Insubria, via JH Dunant 5, Varese, Italy

[2]Department of Biotechnology and Life Sciences, University of Insubria, via JH Dunant 3, Varese, Italy

[3]Department of Pharmaceutical and Pharmacological Sciences, University of Padova, Largo Meneghetti, Padova, Italy

This PDF file includes:

Table S1

Table S2

Table S3

Table S4

Table S1. Intestinal morphology parameters of the proximal and distal gilthead sea bream intestine in the different experimental groups.

**Proximal intestine**		
	**CTRL**	***Hi10***
Villi density	40.5 ± 1.6	35.6 ± 1.23*
Villi height	1444.72 ± 35.2	1406.04 ± 29.57
Villi width	109.76 ± 2.46	99.33 ± 2.88**
Submucosal layer thickness	49.11 ± 1.69	46.53 ± 2.04
Smooth muscle thickness	151.14 ± 5.85	158.13 ± 7.12
Longitudinal muscle thickness	61.6 ± 2.79	66.35 ± 3.57
Circular muscle thickness	91.26 ± 3.32	94.04 ± 3.94
**Distal intestine**		
	**CTRL**	***Hi10***
Villi density	31.8 ± 1.34	31.2 ± 1.07
Villi height	1080.36 ± 40.11	996.56 ± 32.84
Villi width	117.44 ± 3.44	117.2 ± 2.92
Submucosal layer thickness	46.39 ± 2.98	39.88 ± 1.16
Smooth muscle thickness	135.88 ± 5.13	151.36 ± 4.12**
Longitudinal muscle thickness	63.91 ± 2.87	70.74 ± 2.41*
Circular muscle thickness	79.93 ± 2.89	91.61 ± 2.27***

Abbreviations: CTRL: standard fishmeal diet fed fish; Hi10: *Hermetia illucens* 10% meal fed fish. Values are reported as means of ± SEM (n=5 fish). ***$P \leq 0.001$, **$P \leq 0.01$, *$P \leq 0.05$ vs respective CTRL values by Student's t test

Table S2. Frequency and amplitude of spontaneous contractions in the gilthead seabream proximal and distal intestine in the different experimental groups.

Tissue	*Frequency (cicle/sec)*	*Amplitude (g/tissue weight)*
CTRL PI	0.082 ± 0.006	0.29±0.04
Hi10 PI	0.063 ± 0.003*	0.12±0.01***
CTRL DI	0.056 ± 0.006**	0.66±0.11**
Hi10 DI	0.033 ± 0.005§	0.36±0.08§

Abbreviations: PI: proximal intestine; DI: distal intestine; Hi10: *Hermetia illucens* 10% meal. Values are the mean ± SEM; n=5 fish/group. ****P*<0.001 ***P*<0.01 and **P*<0.05 vs CTRL PI; §*P*<0.05 vs CTRL DI by unpaired Student's t test.

Table S3. Ingredients (g/kg) and proximate composition of the experimental diets

Ingredients	CTRL	Hi10
Fish meal	15.0	5.0
Processed animal proteins of poultry	10.7	10.7
Bacterial protein from C. glutamicum	3.9	3.9
Insect meal	0.0	10.0
Soya (bean) meal	10.7	10.7
Guar germ flour	5.0	5.8
Wheat flour	14 .0	14.0
Corn gluten	18.9	18.9
Peas	4.3	3.4
Fish oil	3.9	5.0
Soybean vegetable oil	0.0	0.0
Rapeseed vegetable oil	9.1	7.8
DL-methionine	0.43	0.47
Monoammonium phosphate	0.86	0.86
Lysine HCl	1.29	1.31
Vitamins[a] and minerals[b]	1.15	1.15
Taurine	0.17	0.19
Hydrolyzed fish protein (app)	0.6	0.6
Stay C 35%	0.06	0.06
**Proximate composition**		
Gross Energy (MJ/kg)	19.72	19.67
Digestible energy. DE (MJ/kg)	17.69	17.68
Crude Fat (%)	17.30	17.32
Crude Protein (%)	42.39	42.43
Digestible Protein %	86.53	86.92
Fish Protein (%)	9.00	3.02
Animal Protein (%)	19.09	13.14
FP/TP (%)	21.23	7.11
DP/DE (mg/kJ o g/MJ)	20.73	20.86
AP/TP (%)	45.03	30.97
Fiber (%)	1.07	1.79
NFE (%)	33.47	33.96
Amido (%)	14.26	13.89
NSP (%)	20.28	21.86
Protein-to-lipid ratio	2.45	2.45
Dry Matter (%)	90.8	90.8
Starch In feed (%)	14.3	13.9
Dig. Starch (%)	12.8	12.5
Crude En Starch (%)	586.1	570.7

FP/TP: Fish Protein/Total Protein; DP/DE: Digestible Protein/Digestible Energy, AP/TP: Animal Protein/Total Protein, NFE: Nitrogen-Free Extracts; NSP: Non-Starch Polysaccharides;

[a]Vitamins (mg/kg): Vit. D, 0.05; Vit. A, 2.31 (CTRL), 2.32 (Hi10); Vit. E, 314.55 (CTRL), 315.46 (Hi10); Inositol,

154.26 (CTRL), 154.71 (Hi10); Niacin 189.94 (CTRL), 190.22 (Hi10); Pantothenic ac., 69.34 (CTRL), 69.38 (Hi10); Vit. B2, 27.26 (CTRL), 27.32 (Hi10); Vit. B1, 27.01 (CTRL), 27.05 (Hi10); Vit. B6, 23.10 (CTRL), 23.16 (Hi10); Folic ac. 6.33 (CTRL), 6.35 (Hi10); Vit. K, 5.24 (CTRL), 5.26 (Hi10); Biotin, 0.92 (CTRL), 0.93 (Hi10); Vit. B12, 0.05; Choline, 951.02 (CTRL), 648.55 (Hi10); Vit. C, 211.75.

[b]Minerals: Calcium (% total feed), 1.40 (CTRL), 1.41 (Hi10); Cobalt (mg / kg), 0.29; Copper (mg / kg), 29.72 (CTRL), 29.81 (Hi10); Iron (mg / kg), 310.09 (CTRL), 310.99 (Hi10); Magnesium (% total feed), 20.65 (CTRL), 20.71 (Hi10); Manganese (mg / kg), 8.75 (CTRL), 8.78 (Hi10); Molybdate (mg / kg), 0.34 (CTRL), 0.35 (Hi10); Nickel (mg / kg), 1.03; Phosphorus (% total feed), 0.61 (CTRL), 0.62 (Hi10); Potassium (% total feed), 0.55; Sodium (% total feed), 0.16; Selenium (mg / kg), 0.61 (CTRL), 0.62 (Hi10); Sulfur (% total feed), 0.24; Zinc (mg / kg), 59.47 (CTRL), 59.64 (Hi10).

Table S4. Primary and secondary antisera used and respective dilutions

Antiserum	Dilution	Source	Host species
**Primary antisera**			
HuC/D Biotin	1:100	Invitrogen (16A11)	Mouse
5-HT	1:200	Immunostar (20079)	Goat
Substance P	1:200	Immunostar (20064)	Rabbit
ChAT	1:50	Chemicon (AB144P)	Goat
nNOS	1:50	Thermofisher (61-7000)	Rabbit
β actin	1:100	Cell Signalling (3700S)	Mouse
**Secondary antisera & streptavidin complexes**			
Anti-rabbit Alexa Fluor 488	1:250	Molecular Probes (A21206)	Donkey
Cy3-streptavidin conjugated	1:500	Amersham (PA43001)	
FITC-streptavidin conjugated	1:200	Cedarlane Lab (CLC SA1001)	
Anti-goat Cy3-conjugated	1:500	Jackson IR (705-165-147)	Donkey

Supplying companies: Amersham, GE Healthcare, Buckinghamshire, UK; Cedarlane Inc. Burlington NC, USA; Cell Signalling Danvers, MA, USA; Jackson Immuno Research Laboratories, Inc., Baltimore, USA; Molecular Probes and Invitrogen, Thermo Fisher Scientific, Carlsbad, CA, USA; Chemicon, Merck, Darmastadt, Germany.
